# Ethanolic *Echinacea purpurea* Extracts Contain a Mixture of Cytokine-Suppressive and Cytokine-Inducing Compounds, Including Some That Originate from Endophytic Bacteria

**DOI:** 10.1371/journal.pone.0124276

**Published:** 2015-05-01

**Authors:** Daniel A. Todd, Travis V. Gulledge, Emily R. Britton, Martina Oberhofer, Martha Leyte-Lugo, Ashley N. Moody, Tatsiana Shymanovich, Laura F. Grubbs, Monika Juzumaite, Tyler N. Graf, Nicholas H. Oberlies, Stanley H. Faeth, Scott M. Laster, Nadja B. Cech

**Affiliations:** 1 Department of Chemistry and Biochemistry, University of North Carolina at Greensboro, Greensboro, North Carolina, United States of America; 2 Department of Biological Sciences, North Carolina State University, Raleigh, North Carolina, United States of America; 3 Department of Biology, University of North Carolina at Greensboro, Greensboro, North Carolina, United States of America; University of British Columbia, CANADA

## Abstract

*Echinacea* preparations, which are used for the prevention and treatment of upper respiratory infections, account for 10% of the dietary supplement market in the U.S., with sales totaling more than $100 million annually. In an attempt to shed light on *Echinacea's* mechanism of action, we evaluated the effects of a 75% ethanolic root extract of *Echinacea purpurea*, prepared in accord with industry methods, on cytokine and chemokine production from RAW 264.7 macrophage-like cells. We found that the extract displayed dual activities; the extract could itself stimulate production of the cytokine TNF-α, and also suppress production of TNF-α in response to stimulation with exogenous LPS. Liquid:liquid partitioning followed by normal-phase flash chromatography resulted in separation of the stimulatory and inhibitory activities into different fractions, confirming the complex nature of this extract. We also studied the role of alkylamides in the suppressive activity of this *E*. *purpurea* extract. Our fractionation method concentrated the alkylamides into a single fraction, which suppressed production of TNF-α, CCL3, and CCL5; however fractions that did not contain detectable alkylamides also displayed similar suppressive effects. Alkylamides, therefore, likely contribute to the suppressive activity of the extract but are not solely responsible for that activity. From the fractions without detectable alkylamides, we purified xanthienopyran, a compound not previously known to be a constituent of the *Echinacea* genus. Xanthienopyran suppressed production of TNF-α suggesting that it may contribute to the suppressive activity of the crude ethanolic extract. Finally, we show that ethanolic extracts prepared from *E*. *purpurea* plants grown under sterile conditions and from sterilized seeds, do not contain LPS and do not stimulate macrophage production of TNF-α, supporting the hypothesis that the macrophage-stimulating activity in *E*. *purpurea* extracts can originate from endophytic bacteria. Together, our findings indicate that ethanolic *E*. *purpurea* extracts contain multiple constituents that differentially regulate cytokine production by macrophages.

## Introduction

*Echinacea* is the third most popular herbal medicine in the US, with annual sales of over $100 million [1]. Preparations from several species of this botanical (most commonly *E*. *purpurea* and *E*. *angustifolia*) are used for the treatment of upper respiratory viral infections such as colds and flus, and there is considerable controversy over their efficacy for this purpose (reviewed in [2]). Several clinical trials have demonstrated positive results. For example, Jawad *et al*. [3] treated subjects for 4 months and found a significant reduction in both total number and severity of cold episodes. On the other hand, Barrett *et al*. [4] failed to find an effect when *Echinacea* was used for several days to treat colds once symptoms had emerged. A confounding factor in interpreting the results of clinical trials is the complexity and variability of *Echinacea* products tested. *Echinacea* preparations used in clinical trials have been extracted from different species, different portions of the plant (containing different constituents), and by different processes. In the studies cited above, Jawad *et al*. [3] used a commercial preparation referred to as “Echinaforce”, an ethanol extract of leaves (95%) and roots (5%) of *E*. *purpurea*, while the study conducted by Barrett *et al*. [4] utilized roots from *E*. *purpurea and E*. *angustifolia*, that were ground whole and dispensed in pill form. There is currently a lack of knowledge regarding how to prepare *Echinacea* extracts with specific, desirable biological activities, and which constituents serve as appropriate biomarkers of these activities.

In an attempt to shed light on the mechanism of action of *Echinacea* preparations, their immunodulatory activity has been tested *in vitro*, primarily with studies focusing on macrophages and epithelial cells. During respiratory virus infection, activation of various host pattern recognition receptors (PRRs) on macrophages and epithelial cells leads to the production of pro-inflammatory cytokines, chemokines, and lipids. These molecules in turn bind receptors on a variety of cells types, in a variety of organs and tissues, and trigger the well-known symptoms accompanying respiratory infections including fever, malaise, excess mucus production, and anorexia [5, 6]. For this reason, a number of investigators have hypothesized that the purported relief provided by *Echinacea* extracts arises from inhibition of pro-inflammatory mediator production. *In vitro*, this hypothesis has been tested by adding *Echinacea* extracts to cultures of macrophages and epithelial cells and monitoring effects on production of inflammatory mediators. Unfortunately, the results of these experiments have also been confusing and contradictory. Sharma *et al*. [7] (who tested Echinaforce) found broad inhibition of cytokine production when a number of different respiratory viruses were used to infect epithelial cells and macrophages. Suppression of cytokine and lipid mediator production has also been noted with alkylamides (also referred to as alkamides), which are fatty acid-like constituents of *Echinacea*, following stimulation of macrophages with both respiratory viruses [8] and bacterial LPS [9, 10]. However, not all *Echinacea* extracts suppress cytokine production *in vitro*. In a study where we examined 17 *E*. *purpurea* root extracts (prepared in 75% ethanol), from plants grown on different farms, we found that despite high alkylamide levels, only a few suppressed production of the mediators PGE_2_ and TNF-α from RAW 264.7 cells stimulated with influenza A strain PR/8/34 [8]. In fact, many extracts actually stimulated production of these mediators. Similarly, using aqueous, organic, and detergent-based extraction procedures, Tamta *et al*. [11] and Pugh et. al. [12, 13] found stimulation of soluble mediator production from RAW 264.7 and THP-1 macrophage-like cell lines. These authors attributed this activity to bacterial-derived lipopolysaccharide (LPS) and lipoproteins found in the extracts, and suggested that these compounds originated with endophytic bacteria living in the *Echinacea* plants. Suppression and/or stimulation of cellular activity by *Echinacea* extracts has also been shown with T cells. Sasagawa *et al*. [14] used a 95% ethanol extract of *E*. *purpurea* leaves and flowers and demonstrated inhibition of phytohemagglutinin-dependent production of IL-2 with the Jurkat human T cell line, a finding that was subsequently linked to the inhibition of the nuclear receptor PPARγ[15]. More recently, using an aqueous *E*. *purpurea* extract with high polysaccharide content, Fonseca *et al*. [16] also reported inhibition of Jurkat T cell function at low cell densities. However, these authors also reported stimulation of cell function by this extract when Jurkat cells were grown at higher cell densities [16]. The reason for this discrepancy is unclear.

Our laboratories are interested in understanding the *in vitro* effects of *Echinacea* extracts and their constituents. Can these models be used ultimately to predict activity *in vivo*? The use of different extracts by different laboratories, yielding complex and confusing *in vitro* results, suggested to us that we take a step backward and more carefully dissect the chemical basis for the *in vitro* immunomodulatory activity of a single extract. Insights gained from such experiments should then be useful to inform rational design of *Echinacea*-based therapies *in vivo*. In these experiments, we hypothesized that complex *Echinacea* extracts contain complex mixtures of molecules, both of botanical and bacterial origin, capable of differentially modulating immune cell function. Our goal with these studies was to test this hypothesis by comparing the effects of an *E*. *purpurea* (L.) Moench (Asteraceae) ethanolic root extract, and its constituents and fractions, on the production of cytokines and chemokines from the RAW 264.7 macrophage-like cell line. We also sought to explore the potential role of LPS from endophytic bacteria in dictating the *in vitro* immunomodulatory activity of *E*. *purpurea* extracts. We focused specifically on *E*. *purpurea* in these studies because this is the *Echinacea* species that has been most widely used for medicinal purposes in the US [17].

## Materials and Methods

### Instrumentation

Flash chromatography separations were performed using an automated Isco CombiFlash RF system over silica gel columns (Teledyne Isco, Lincoln, NE). High performance liquid chromatography (HPLC) separations were accomplished using a Varian HPLC system (ProStar 210 pumps, ProStar 710 fraction collector, ProStar 335 photodiode array detector) with Galaxie Chromatography Workstation software (version 1.9.3.2). An Acquity ultra-high performance liquid chromatography (UHPLC) system (Waters Corporation, Milford, MA) coupled to a LTQ Orbitrap XL Hybrid mass spectrometer (Thermo Fisher Scientific, Waltham, MA) was used for all LC-MS analyses. NMR spectra were acquired with a JNM-ECS 400 MHz NMR spectrometer (JOEL USA, Peabody, MA). Unless otherwise stated, all solvents used in chemical analyses were purchased from Thermo Fisher Scientific (Waltham, MA).

### Preparation and fractionation of a large scale *E*. *purpurea* root extract

**Fig 1:**
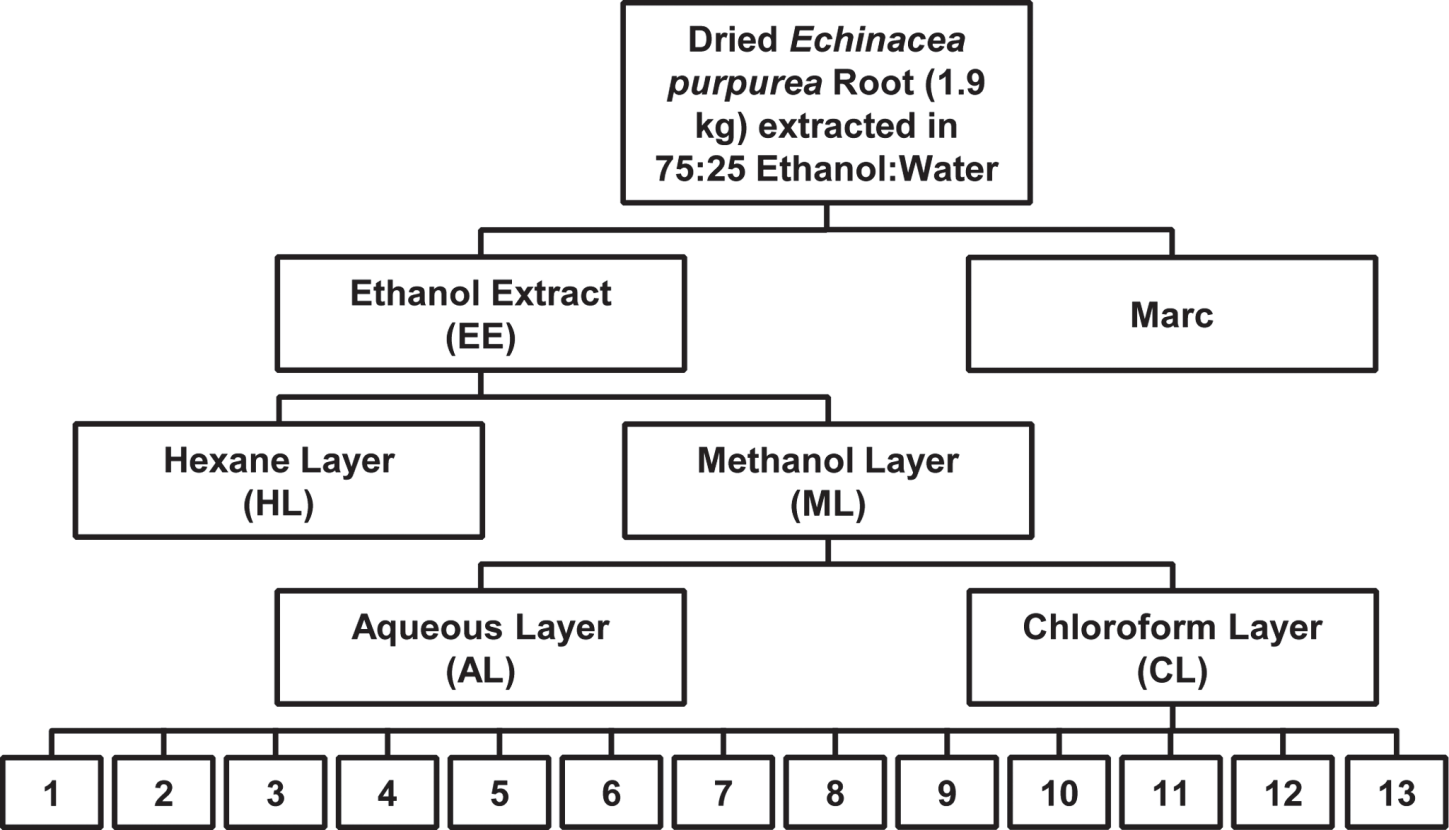
Partitioning scheme for the *Echinacea purpurea* extract. Dried *E*. *purpurea* roots (1.9 kg) were extracted in 75:25 ethanol:water, consistent with standard practices in the dietary supplements industry. The ethanolic extract was evaporated by rotary evaporation and subjected to two stages of liquid:liquid partitioning with hexane:methanol and chloroform. The resulting chloroform layer was fractioned into thirteen pooled fractions using normal phase flash chromatography. These fractions were then profiled for chemical composition and subjected to biological assays. Marc, residue after extraction; EE, ethanol extract; HL, hexane layer, ML, methane layer; AL, aqueous layer; CL, chloroform layer.

*Echinacea purpurea* roots were purchased from Pacific Botanicals (Grants Pass, OR). A voucher specimen (NCU 633811) was deposited at the North Carolina Herbarium. Roots were rinsed, crudely cut, and allowed to dry at 37°C for 1 week. Dried root material was ground mechanically using a Wiley Mill Standard Model No. 3 (Arthur H. Thomas Co., Philadelphia PA) to mesh size 2 mm. The ground root material (1.9 kg) was macerated for seven days in 75% ethanol (Pharmaco-AAPER, Shelbyville, KY) at a ratio of 5:1 (mL of solvent:g of dried root material). The resulting extract was vacuum filtered using Whatman 24.0 cm grade 1 filter paper, and the filtered extract was concentrated using a rotatory evaporator and subjected to two stages of liquid-liquid partitioning (Fig 1). The first stage consisted of defatting by partitioning between hexane and 10% aqueous methanol (1:1). Layers were separated and dried using a rotatory evaporator. The dried aqueous methanol layer was then partitioned using water:methanol:chloroform (4:1:5), resulting in two layers that were separated and again subjected to rotary evaporation. The chloroform layer was fractionated with normal-phase flash chromatography over a RediSep Rf silica gel column using a gradient of 100% hexane to 100% chloroform to 100% methanol. The column eluent was combined into 13 pooled fractions based on LC-UV chromatograms and the fractions were evaporated and stored dry at 4°C until needed for bioassay or chemical analysis.

### Isolation and identification of xanthienopyran

Fractions 9 and 10, prepared as described in the previous section, were combined based on similar anti-inflammatory activity and LC-MS profile for a total yield of 747.7 mg. This combined sample was subject to normal-phase flash chromatography using a RediSep Rf silica gel column and a hexane:chloroform:methanol solvent system at a flow rate of 40 mL/min. The gradient initiated at 50:50 (hexane:chloroform) and increased to 100% chloroform over 3 column volumes, was held at 100% chloroform for 3 column volumes, decreased linearly to 10:90 (methanol:chloroform) over 5 column volumes, then decreased linearly to 20:80 (methanol:chloroform) over 10 column volumes, decreased linearly to 50:50 (methanol:chloroform) over 4 column volumes, decreased to 100% methanol over 2 column volumes and was held at 100% methanol for 2 column volumes. Fractions were combined into 8 pooled fractions (A-H) based on UV absorbance.

Fraction E eluted from column after 14 column volumes and yielded 392.9 mg. This fraction was subjected to an additional stage of flash chromatography with a silica gel Gold column using a hexane:ethyl acetate:methanol solvent system and a flow rate of 30 mL/min. The gradient began at 50:50 (hexane:ethyl acetate) and increased to 100% ethyl acetate over 2 column volumes, continued isocratically at 100% ethyl acetate for 5 column volumes, decreased linearly to 10:90 (methanol:ethyl acetate) over 5 column volumes, continued to decrease linearly for 10 column volumes to 20:80 (methanol:ethyl acetate), decreased to 100% methanol over 2 column volumes, and was held at 100% methanol for 2 column volumes. The eluent was combined into 3 pooled fractions (I-III) based on UV absorbance.

Fraction I contained eluent from the first three column volumes (yield: 174.9 mg) and was subject to further separation using HPLC with a Gemini-NX 5 μm, 250 × 21.2 mm C-18 column (Phenomenex Inc., Torrance, CA). Samples were eluted from the column using a binary solvent system with solvent A consisting of water (Barnstead Nanopure Diamond, Thermo Fisher) with 0.1% formic acid and solvent B consisting of acetonitrile (HPLC grade) with 0.1% formic acid at a flow rate of 21 mL/min. The gradient began at 60:40 (A:B) and decreased linearly to 20:80 (A:B) over 20 min, then decreased linearly to 0:100 (A:B) over 5 min. and was held at isocratic conditions for 5 min. The eluent was combined into three pooled fracitons (a-c) based on UV absorbance. The second fraction (b) yielded 3.2 mg of 95% pure xanthienopyran (**1**). NMR and high resolution LC-MS were employed to confirm the identification of the isolated compound.

### Quantitative analysis of alkylamides

Alkylamide concentraitons were determined using LC-MS. Each sample was re-suspended in methanol to a concentration of 1 mg/mL prior to analysis, and serial dilutions of these solutions were performed to achieve a concentration within the linear range of the calibration curve. A 3 μL injection of each sample was eluted from the column (Acquity UPLC BEH C18 1.7μm, 2.1 × 50 mm, Waters Corp.) at a flow rate of 0.25 mL/min using the following binary gradient with solvent A consisting of water (Optima LC/MS grade) with 0.1% formic acid additive and solvent B consisting of acetonitrile (Optima LC/MS grade) with 0.1% formic acid additive. The gradient initiated at an isocratic composition of 90:10 (A:B) for 1.0 min, increasing linearly from 1.0–8.0 min. to 10:90 (A:B), followed by an isocratic hold at 10:90 (A:B) from 8.0–9.0 min, gradient returned to starting conditions of 90:10 (A:B) from 9.0–9.1 min, and was held at this composition from 9.1–10 min. The mass spectrometer was operated in positive ionization mode over a scan range of 150–1000 with the following setting: capillary voltage set at 5 V, capillary temperature set at 300°C, tube lens offset set at 35 V, spray voltage set at 3.80 kV, sheath gas flow set at 35, and auxiliary gas flow set at 20.

Alkylamides were identified by comparing retention times and *m/z* values with previously published literature values [18], as described in detail elsewhere. The selected-ion chromatograms for the six most abundant alkylamides were plotted, and the peak area was determined for each. These peak areas were used to compare the alkylamide concentration within each of the fractions. One of the abundant alkylamides in the 75% ethanol extracts, dodeca-2E,4Z-diene-8,10-diynoic acid isobutylamide (**5**), was quantified by linear regression of an external calibration curve generated using an dodeca-2E,4Z-diene-8,10-diynoic acid isobutylamide (**5**) standard (Chromadex, Irvine CA) (Table 1). Concentrations for the remaining 5 alkylamides was estimated using the regression parameters for the dodeca-2E,4Z-diene-8,10-diynoic acid isobutylamide calibration curve.

**Table 1:** Alkylamide content of an *Echinacea purpurea* extract, chloroform layer, and column fractions from the chloroform layer.

Sample	Concentration of alkylamide 5 ± SD (μg/mg extract)^a^	Estimated total alkylamide content ± SD (μg/mg extract)
95% ethanol extract	7.9 ± 0.57	51 ± 8.2
Chloroform layer	26 ± 1.1	140 ± 4.7
Fraction 1	-	-
Fraction 2	Below LOQ^b^	Below LOQ
Fraction 3	ND^c^	ND
Fraction 4	ND	ND
Fraction 5	ND	ND
Fraction 6	63 ± 12	310 ± 42
Fraction 7	ND	2.2 ± 0.38
Fraction 8	ND	0.14 ± 0.034
Fraction 9	Below LOQ	Below LOQ
Fraction 10	ND	ND
Fraction 11	ND	ND
Fraction 12	ND	ND
Fraction 13	ND	ND

a. Concentration is reported for one of the major alkylamides in the extract, alkylamide 5 in Fig 3 (dodeca-2E,4Z-diene-8,10-diynoic acid isobutylamide). The total alkylamide concentration was estimated using the calibration curve for this compound. Reported concentrations are means+/- SD from triplicate measurements by LC-MS as described in the Materials and Methods.

b. Below LOQ (limit of quantification) indicates that alkyamides were detected but were present at concentrations below the limit of quantitation for the method.

c. ND indicates that alkylamides were not detected, i.e. not present at levels above the limit of detection (LOD).

### Quantitative analysis of lipopolysaccharides

To quantify lipopolysaccharide (LPS) levels in the extracts from sterilized *E*. *purpurea* plants, a small amount (0.16–19.55 mg) of each was re-suspended in 1 mL of ethanol (PHARMACO-AAPER, USA). Each suspension was then diluted 1:1000 with endotoxin free water, and LPS was quantified using the Chromo-LAL assay (Associates of Cape Cod Inc., Falmouth, MA). Reagents were provided by Associates of Cape Cod Inc. and analyses were performed on a Synergy H4 Microplate Reader (BioTek Instruments, Inc, Winooski, VT).

### Growth and extraction of *Echinacea purpurea* under aseptic conditions

*E*. *purpurea* seeds were obtained from Horizon Herbs, LLC (lot # 6784, Williams, OR) and a voucher for the whole *E*. *purpurea* plants form this source is on file at the North Carolina Herbarium (NCU583422). Two methods were used to sterilize seeds prior to germination. With the first method, designed to remove surface bacteria but not bacterial endophytes (bacteria living inside the seeds), the achene pericarp was removed, and the seeds were subjected to a surface sterilization procedure slightly modified from that reported by Leuchtmann and Clay [19]. Sterilization was accomplished by sequential soaking for 1 min. in ethanol (70%), followed by 4 min. in sodium hypochlorite solution (~4.5%), and finally 30 sec in ethanol (70%). The seeds were then rinsed in sterile water and germinated as described below.

A second sterilization procedure was used to kill both surface and endophytic bacteria. This process included an added step of removal of the seed epidermis prior to sterilization. To remove the epidermis, pericarp-free seeds were soaked in a water bath for 20 min. at 40°C, and individually scrubbed on a paper towel to disrupt the unicellular epidermis layer. Epidermis fragments were removed with forceps. These seeds were then subjected to the same sterilization method described above for the epidermis-intact seeds.

Germination was induced on water agar Petri dishes (1.5%) with seven seeds per plate. The seeds were monitored daily for outgrowth of bacteria or fungi, and all seeds or seedlings with signs of such contamination were excised and removed from the agar plate. After seven days, all remaining symptomless seedlings were planted into sterile cultivation containers (Plantcon, MP Biomedicals, LLC, Solon, OH), which contained 150 mL agar with Murashige Skoog media [20] adjusted to pH = 7 and were maintained in sterile conditions at 25/25°C and 12 hr day/night cycle in a climate chamber (Adaptis A1000, Conviron, Pembina, ND) until harvest.

Individual *E*. *purpurea* plants were harvested in a laminar flow hood, and transferred to sterile 100 mL glass bottles, lyophilized for 48 hr, and stored at -80°C until extraction. Each plant was extracted in 8 mL of 75% ethanol. Extracts were filtered using a 0.2 μm puradisc filter (Whatman, GE Healthcare UK Limited, UK) connected to a nonpyrogenic syringe (Henke Sass Wolf, Germany). An aliquot (1 mL) of each extract was diluted 1:1000 with water and analyzed using the Chromo-LAL assay as previously described. An additional 1 mL aliquot of each plant extract was evaporated to dryness in a preweighed vial to determine mass of plant material per volume of extract.

### Cell culture

Ethanol was used as the vehicle for *E*. *purpurea* extracts, partitions and fractions in all cell culture experiments. The maximum ethanol concentration was 1% in all assays and was found not to affect cytokine production. RAW 264.7 cells were obtained from the American Type Culture Collection, USA and were cultured in Dulbecco's modification of Eagle’s medium (DMEM) supplemented with 4 mM L-glutamine, 4.5 g/L glucose, and 3.7 g/L sodium bicarbonate, 10% fetal bovine serum (FBS) at 37°C with 5% CO_2_. Media and supplements were obtained from Caisson Labs (Logan, UT) and Sigma Aldrich (St. Louis, MO). FBS was obtained from Gemini Bio-Products (Sacramento, CA).

### Cell treatment and cytokine measurements

For bioassay of crude extracts and partition layers, RAW 264.7 cells were seeded into 24-well tissue culture plates (Genesee Scientific, USA) at a density of 1.5 x 10^5^ cells/well and allowed to adhere for 24 hr. Extract, partition layers, and fractions were added at indicated concentrations. The extracts prepared from endophyte-free plants were tested by adding 6.7 μL directly to the cell culture medium under the conditions described previously [8]. For analysis of column fractions, RAW 264.7 cells were seeded into 96-well plates at 2.5 x 10^4^ cells/well. Treatments included each fraction or starting material alone or in combination with 10 or 100 ng/mL of LPS from *Salmonella minnesota* R595 (List Biological Laboratories, USA) for 16–18 hr. Supernatants were collected, centrifuged at 16,000 x g for 5 min. and stored at -80°C until analysis. TNF-α, CCL3/MIP-1α and CCL5/RANTES ELISA kits were purchased from R&D Systems, USA or eBioscience, USA. Optical density was determined using a Synergy HT microplate reader (BioTek Instruments, Inc.). Cytokine concentrations were interpolated from standard curves.

### Cytotoxicity assay

RAW 264.7 cells were seeded into 96-well plates and treated with each fraction or starting material alone under the conditions described previously. Supernatants were collected after an 18 hr. incubation and analyzed for lactate dehydrogenase (LDH) activity using a commercially available kit according to the manufacturer’s instructions (Thermo Fisher Scientific). Briefly, in a 96-well plate, 50 μL of supernatant was incubated with 50 μL of reaction mixture containing a tetrazolium salt that is reduced to formazan in the presence LDH. The plate was incubated for 30 min. protected from light before 50 μL of stop solution was added. The absorbance was read at 490 nm with absorbance at 690 nm subtracted to remove background. Vehicle treated cells were lysed with lysis buffer and used as the maximum LDH release.

### Statistical analyses

Significant differences between means were determined using the Student’s T test with GraphPad Prism software (GraphPad Software, San Diego, CA). Levels of significance are indicated in individual figure legends.

## Results and Discussion

The broad objective of these studies was to develop a more complete understanding of the activity of an *E*. *purpurea* extract, and its constituents, on the production of cytokines and chemokines by macrophages. To achieve this goal, we utilized the RAW 246.7 murine macrophage-like cell line as a model, rather than primary mouse macrophages. With the use of this cell line, it was possible assay the large number of samples produced as a result of these experiments. RAW 264.7 cells have been used extensively in studies of macrophage cytokine production, and, like primary murine or human macrophages, produce an array of inflammatory cytokines and chemokines following stimulation with agonists such as LPS. We have shown that these cells produce high levels of six cytokines and chemokines following LPS stimulation, including TNF-α, IL-6, G-CSF, CCL2 (MCP-1), CCL3 (MIP1-α), and CCL5 (RANTES) [21]. In the experiments described in this manuscript, we focused largely on RAW 264.7 cell production of TNF-α. TNF-αas was selected because it is a key inflammatory cytokine whose overproduction has been linked to several chronic and acute inflammatory disorders such as inflammatory bowel disease [22], psoriasis [23], and rheumatoid arthritis [24]. We and others have shown that *Echinacea* extracts are capable of modifying TNF-α production [7, 21] and a more complete understanding of these effects may lead to useful treatments for disease. In our experiments, we also sought to obtain a broader view of the impact of *E*. *purpurea* extracts on RAW 264.7 cell secretory activity, and therefore we expanded our analysis to include two of the chemokines mentioned above (CCL3 and CCL5). Finally, we tested each *E*. *purpurea* extract or fraction using two complementary approaches. The ability of the samples to induce the production of inflammatory cytokine and chemokines production was evaluated by adding them directly to RAW 264.7 cells in media with appropriate solvent controls. Alternatively, the inhibitory activity of the samples on production of inflammatory cytokines and chemokines was tested by adding them to RAW 264.7 cells that had been activated by the addition of LPS.

### Extract preparation and influence on cytokine secretion

**Fig 2:**
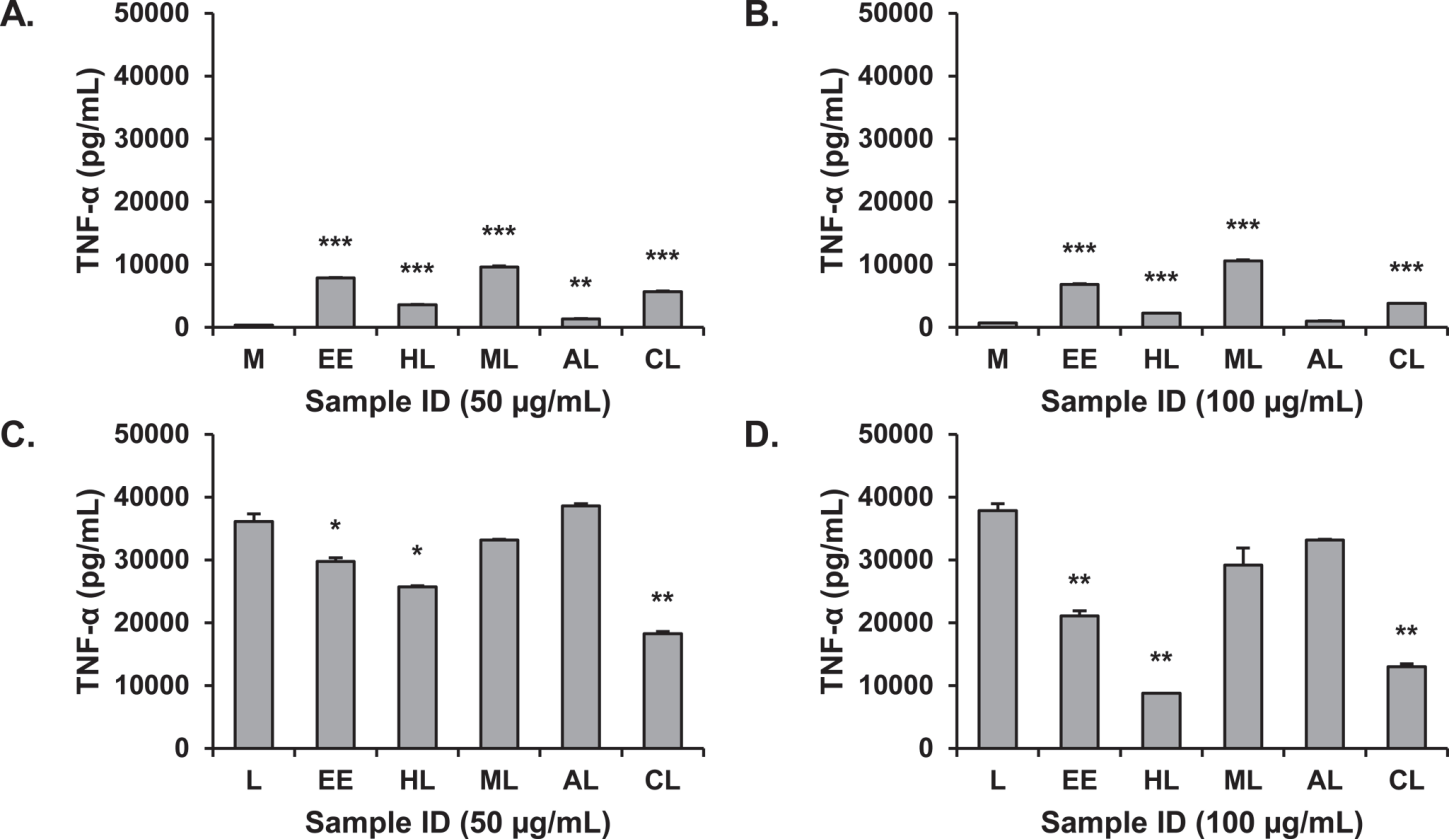
Influence of 75% ethanol extract and liquid:liquid partitions from *Echinacea purpurea* extract on TNF-α production by RAW 264.7 cells. Extract and partitions were tested at concentrations of 50 μg/mL (A and C) and 100 μg/mL (B and D) expressed as mass of extract per assay well volume. Cells were unstimulated in A and B and stimulated with 100 ng/mL LPS in C and D. Supernatants were harvested after 16–18 hr. and levels of TNF-α measured by ELISA. M, media; L, LPS; EE, ethanol extract; HL, hexane layer, ML, methane layer; WL, water layer; CL, chloroform layer. Values shown are means +/- SD from a single representative experiment. Statistical analysis was performed using the Student’s T test, *p<0.05, **p<0.01, ***p<0.001.

Our studies employed an *E*. *purpurea* extract that was prepared consistent with practices commonly employed in the dietary supplements industry. *E*. *purpurea* roots were collected, dried, and extracted in 75% ethanol. The ethanol extract (EE) was then tested *in vitro* for its effects on production of TNF-α from RAW 264.7 cells. This extract reproducibly induced the production of TNF-α (Fig 2), with 10 ng/mL typically accumulating in cultures with a 24 hr treatment. This amount was similar when concentrations of both 50 and 100 μg/mL of the extract were tested (Fig 2A and 2B), suggesting that the maximum level of stimulation had been reached. From a comparative perspective, the level of TNF-αinduced by the extract was not very high for these cells under these culture conditions. For example, as shown in Fig 2C and 2D, levels of TNF-α can exceed 30 ng/mL when a dose of 100 ng/mL is used to stimulate RAW 264.7 cells.

The ethanolic extract (EE) was also tested for its ability to inhibit production of TNF-α in the presence of LPS (Fig 2C and 2D). At a concentration of 50 μg/mL, it displayed a significant but weak suppressive activity of approximately 10–20% (Fig 2C). However, at the higher, 100 μg/mL concentration, the suppressive effect was much more pronounced, approaching 50% (EE, Fig 2D). Apparently, the molecules mediating the suppressive effect were not present at sufficient concentration when the extract was tested at 50 μg/mL, and the higher 100 μg/mL concentration was necessary to observe the suppressive effect. It is likely that this activity would have been overlooked had we not tested higher extract concentrations. We concluded, therefore, that the complex ethanolic *E*. *purpurea* extract contains molecules capable of both stimulating and inhibiting macrophage secretion of TNF-α and that it is critical to examine a range of extract concentrations to detect these activities *in vitro*. It is also clear that the net effects of this extract to either stimulate or suppress production of TNF-α should be thought of as the sum of multiple, possibly opposing activities rather than as a single stimulatory or suppressive activity. In the future, it will be interesting to determine whether both activities are bioavailable *in vivo* and whether they offset each other’s effectiveness.

### Fractionation and alkylamide content of the extract

Alkylamides from *E*. *purpurea* are well known to suppress production of inflammatory mediators *in vitro* [8, 9, 25], and are used as markers for standardization of some *E*. *purpurea* preparations. Thus, we sought to determine if the ability of the complex ethanolic *E*. *purpurea* extract to suppress secretion of TNF-α could be attributed to the presence of alkylamides. Quantitative analysis of the alkylamide content of the ethanolic *E*. *purpurea* extract indicated that it contained approximately 51 μg/mg (5.1%) total alkylamides, which corresponds to an assay concentration of 2.6 μg/mL alkylamides in Fig 2C. Previously, a higher concentration of alkylamides has been reported to be necessary to significantly suppress TNF-α secretion by RAW 264.7 cells (6–12 μg/mL) [8]. Thus, we suspected that components besides alkylamides contributed to the activity of the crude *E*. *purpurea* extract. To investigate this further, the extract was partially purified with two stages of liquid-liquid partitioning (Fig 1), and each of the resulting layers was tested for its ability to induce or inhibit the production of TNF-αby RAW 264.7 cells. As shown in Fig 2A and 2B, the stimulatory activity was generally conserved in the methanol and chloroform layers (ML and CL, respectively). The chloroform layer also demonstrated significant suppressive activity, as did the hexane layer (HL) (Fig 2C and 2D).

**Fig 3:**
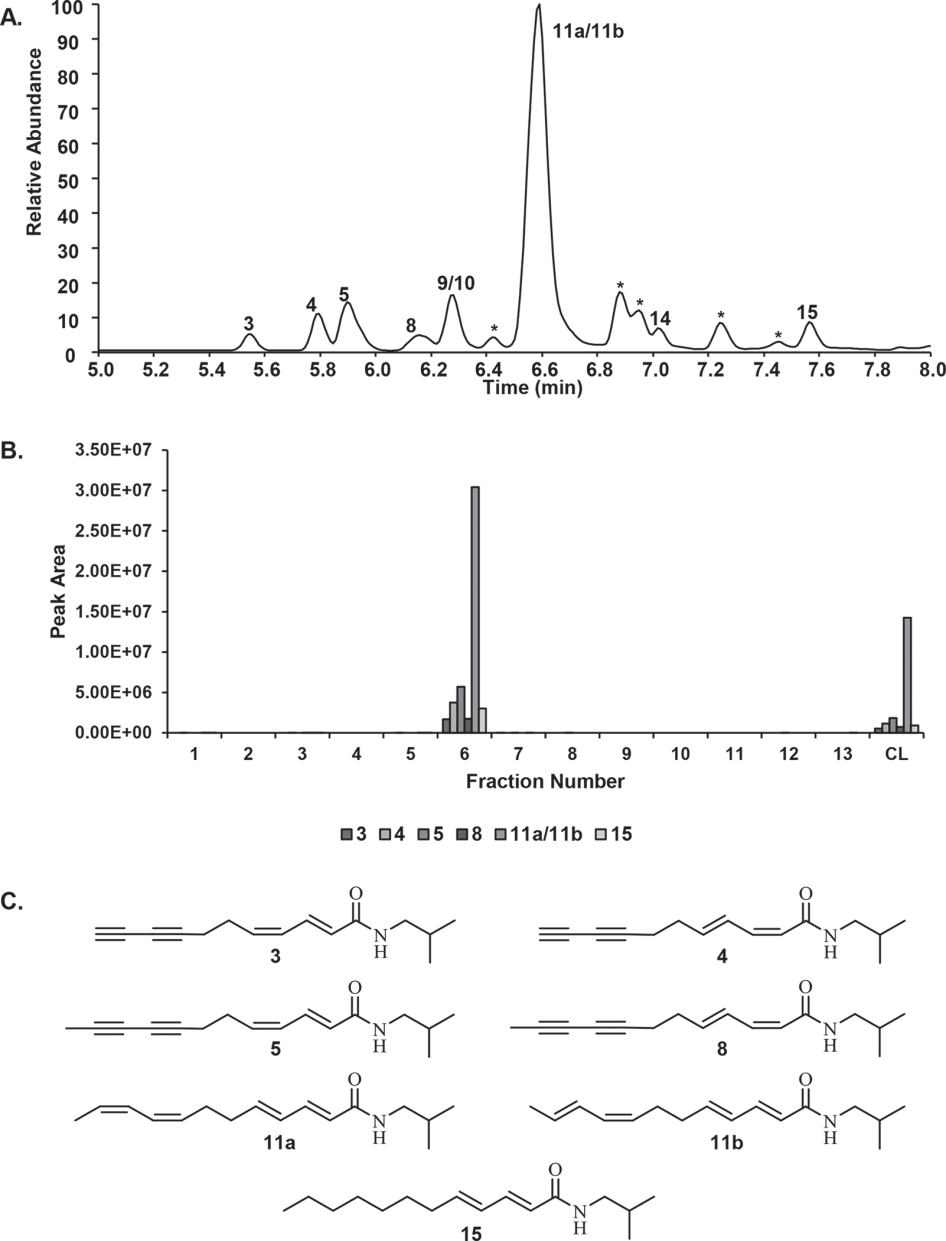
Alkylamides from *Echinacea purpurea*. Chromatogram (A) of the crude chloroform layer (CL) from the *E*. *purpurea* extract showing the presence of a series of alkylamides (3,4,5,8,9,10,11A,11B,14,15) which were identified according to molecular weight and retention time. The relative amounts of these alkylamides in the silica gel column pooled fractions (1–13) are indicated by the peak areas shown in panel B. The numbering system used for these alkylamides is consistent with that used in a previous report [18]. * indicates compounds detected, but not identified. The structures for seven alkylamides selected to represent the alkylamide content are shown in panel C.

An additional level of fractionation, using flash chromatography over silica gel (Fig 1), was undertaken to further purify the components responsible for the cytokine-suppressive activity of the *E*. *purpurea* extract. The chloroform layer was chosen as starting material for these fractionation efforts, but it is notable that additional anti-inflammatory constituents could be present in the hexane layer (HL), which will be the focus of future studies. The chloroform layer was separated into thirteen fractions, and the original chloroform layer and each fraction was analyzed for alkylamide content. *E*. *purpurea* can produce an array of alkylamides that differ in chain length and number and placement of double and triple bonds in the fatty acid portion of the molecule [26]. Analysis of the chloroform layer indicated the presence of a number of structurally distinct alkylamides (Fig 3). Consistent with previous reports [8], alkylamide **11a/b** was the dominant alkylamide. The alkylamide numbering system used here has been published previously [18], and can be cross-referenced with that used by Bauer [26].

As a result of the partitioning process, the alkylamide content of the chloroform layer was increased 2.7-fold as compared to that of the ethanol extract, for a total alkylamide content of 140 ± 4.7 μg/mg (Table 1). Similarly, fractionation of the chloroform layer concentrated the alkylamides into fraction 6, which contained 310 ± 42 μg/mL alkylamides (Table 1), a 2.2-fold increase. Thus, the chloroform layer and fraction 6 contained alkylamides within the concentration range previously demonstrated to be biologically active [8], assay concentrations of 7.0 μg/mL and 15.5 μg/mL total alkylamides, respectively, for the experiment reported in Fig 2C.

**Fig 4:**
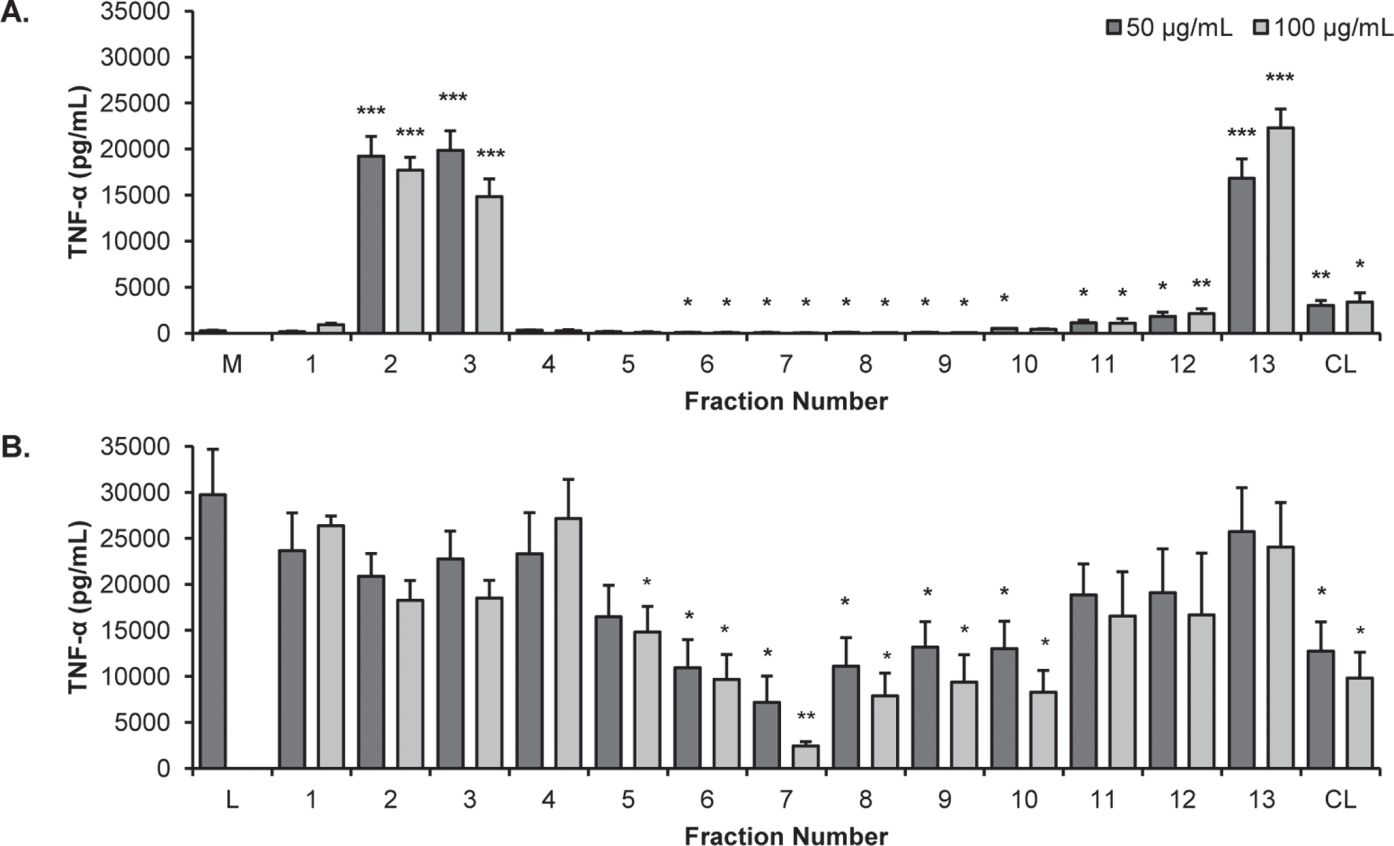
The effects of *Echinacea purpurea* extract fractions on production of TNF-α. Fractions from the flash chromatography separation of the *E*. *purpurea* extract chloroform layer (CL) were tested for their effects on the production of TNF-α from RAW 264.7 cells in the absence (A) or presence (B) of 10 ng/mL LPS. Treatments were for 16–18 h and levels of TNF-α in supernatants were quantified by ELISA. Each fraction was tested at 50 and 100 μg/mL. All data shown are means +/- SEM from three independent experiments. Statistical analysis was performed using the Student’s T test, *p<0.05, **p<0.01. M, media; L, LPS; CL, chloroform layer.

Testing with RAW 264.7 cells revealed that three of the fractions (2, 3 and 13) strongly stimulated the production of TNF-α (Fig 4A). In contrast, production of TNF-α was significantly inhibited by fractions 6–10 (Fig 4B). These results further substantiate the presence of both stimulatory and inhibitory activities present in the original *E*. *purpurea* extract, and indicate that chemical manipulations can be used to separate these activities from each other. It may be possible, therefore, to use standard chemical processes to produce extracts with, for example, enhanced suppressive activity.

Consistent with our earlier prediction, the results in Fig 4B indicate that the alkylamides are not solely responsible for the suppressive effects noted in the chloroform layer. As expected, the alkylamide-rich fraction 6 did suppress the LPS-induced production of TNF-α but several other fractions (7–10) exerted this effect as well. The suppressive effects of these fractions must be explained by the presence of other compounds. Other bioactive small molecules previously reported as constituents of *E*. *purpurea*, such as the phenolic compounds caftaric acid and cichoric acid [27], and various ketone derivatives [10], could play a role in this activity. Overall, however, we suspect that the TNF-α suppressive activity of *E*. *purpurea* extracts is due to the collective action of a host of small molecules, many of which have not yet been identified.

### Cytotoxicity

**Fig 5:**
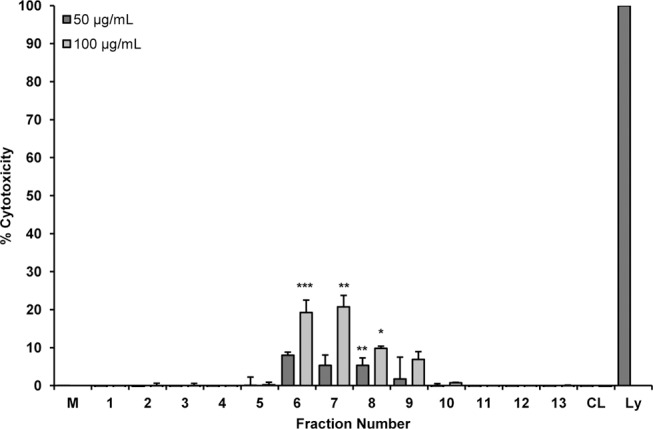
Cytotoxicity of *Echinacea purpurea* extract fractions and chloroform layer. Fractions from the silica gel column and the chloroform layer were tested for cytotoxicity towards RAW 264.7 cells. Treatments were for 24 h and cytotoxicity was determined using the LDH release assay. Each fraction was tested at 50 and 100 μg/mL. Data shown are means +/- SEM from three independent experiments. Statistical analysis was performed using the Student’s T test, *p<0.05, **p<0.01, ***p<.001. M, media; CL, chloroform layer; Ly, lysis buffer.

In the experiments presented herein (Figs 2 and 4), we noted suppression of TNF-α production, which could occur if the added samples cause cell death. Each sample was therefore tested for lactate dehydrogenase (LDH), a cytosolic enzyme released from dying cells into the culture supernatant. As shown in Fig 5, the chloroform layer and most column fractions did not display any cytotoxic effects. A low level of cytotoxicity was noted for fractions 6–9, 1–5% at 50 μg/mL and 10–25% at 100 μg/mL, respectively. Although cell toxicity may contribute somewhat to the observed activity of these fractions, it is highly unlikely that they account for the observed 50–90% inhibition of TNF-α (Fig 4).

### Identification of xanthienopyran

**Fig 6:**
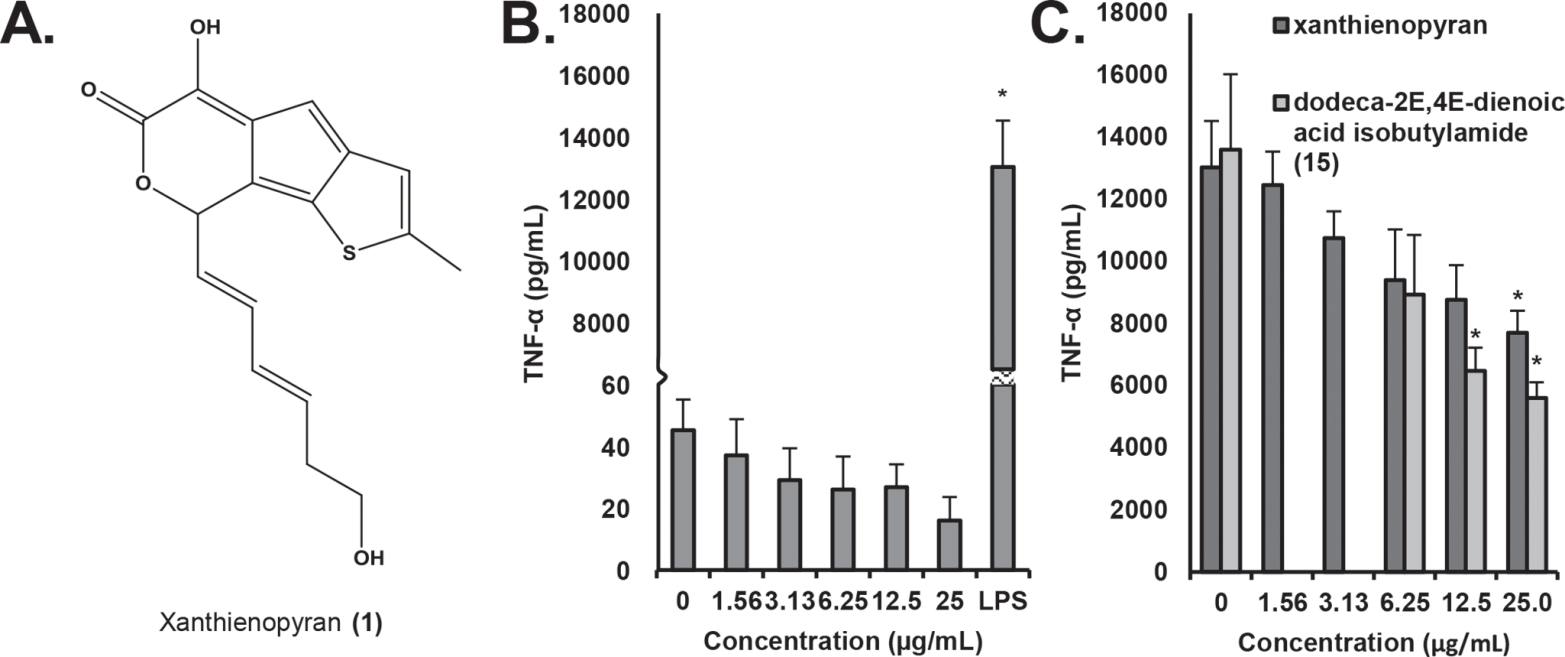
Structure and activity of xanthienopyran. The structure of xanthienopyran was elucidated by comparison of NMR and MS data with published values (A). The influence of xanthienopyran on TNF-α secretion by RAW 264.7 macrophage-like cells alone (B) or in the presence of 10 ng/mL of LPS (C) was evaluated. The influence of dodeca-2E,4E-dienoic acid isobutylamide (**15**) on TNF-α secretion by RAW 264.7 macrophage-like cells in the presence of 10 ng/mL of LPS (C) was evaluated as comparison. Treatments were for 16–18 hr. and levels of TNF-α in supernatants were quantified by ELISA. Data shown are means +/- SEM from three independent experiments. Statistical analysis was performed using the Student’s T test, *p<0.05.

In an attempt to identify other compounds that play a role in the anti-inflammatory activity of *E*. *purpurea*, the column fractions that significantly suppressed production of TNF-α from LPS-stimulated cells but did not contain high alkylamide content (7–10) were subjected to further purification. Several of these fractions (7 and 8) were found to be very complex and/or of very low yield, and efforts to isolate pure compounds from them were unsuccessful. However, the compound xanthienopyran (**1**) (Fig 6A) was isolated from combined fractions 9 and 10. Xanthienopyran has previously been reported as a constituent of the fruit and seeds of various *Xanthium* species [28], but this is the first report of this compound from *E*. *purpurea*. NMR data for xanthienopyran (S1 Fig) were in agreement with those previously published [29]. Additionally, the measured monoisotopic mass ([M+H]^+^ = 317.0838) was within 2.9 ppm mass error of the calculated mass ([M+H]^+^ = 317.0847).

Xanthienopyran has been shown to inhibit the production of superoxide anions in human neutrophils [28], but its influence on macrophages has not previously been evaluated. We found that xanthienopyran itself did not stimulate production of TNF-α (Fig 6B). However, it caused a dose dependent inhibition of the LPS-induced production of TNF-α (Fig 6C). From these data, an IC_50_ of 3.4 μg/mL was calculated, very similar to the IC_50_ of 1.7 μg/mL reported for inhibition of superoxide production from neutrophils [28]. Taken together, our data suggest that alkylamides and xanthienopyran contribute to, but do not entirely explain, the activity of the *E*. *purpurea* extract to suppress the LPS-induced production of TNF-α.

### The broad nature of the suppressive effect

**Fig 7:**
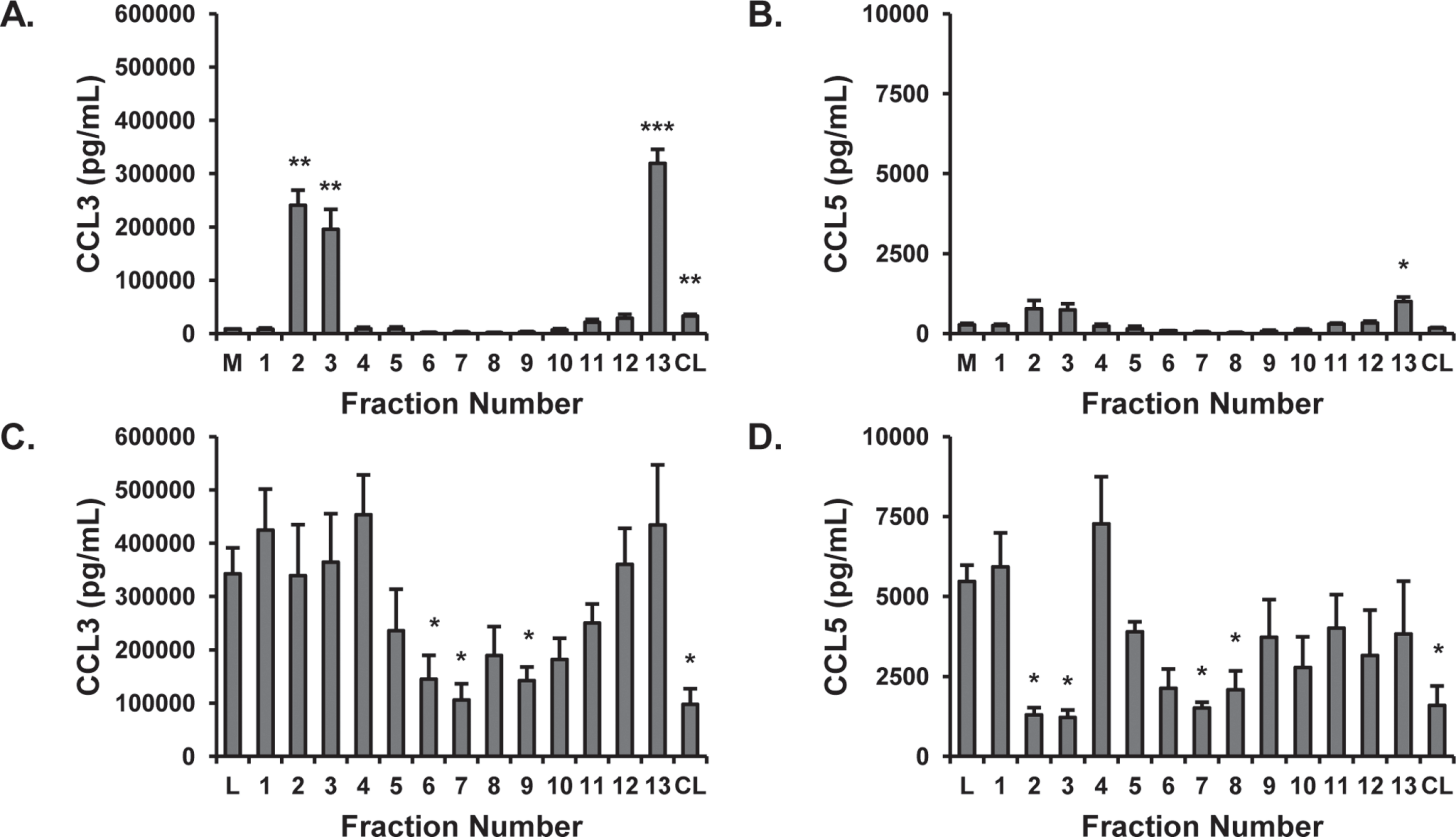
*Echinacea purpurea* fraction effects on CCL3 and CCL5. Fractions from the silica gel column were tested for their effects on the production of CCL3 (MIP1-α) and CCL5 (RANTES) from RAW 264.7 cells in the absence (A and B) or presence (C and D) of 10 ng/mL LPS. Treatments were for 16–18 hr. and chemokine levels in supernatants were quantified by ELISA. Each fraction was tested at 50 μg/mL. Data shown are means +/- SEM or SD from three (CCL3) and two (RANTES) independent experiments. Statistical analysis was performed using the Student’s T test, *p<0.05. M, media; CL, chloroform layer.

The experiments described thus far have focused on TNF-α as an indicator of macrophage mediator production. However, this cytokine is only one of several cytokines and chemokines produced by RAW 264.7 cells following treatment with LPS or other pathogen associated molecular patterns. Previously, we [8] and others [7, 9] have performed comprehensive studies of *Echinacea* extracts and their effects on macrophage secretory products, and this was not our goal in this investigation. It was of interest, however, to evaluate whether the activities we were monitoring were specific for TNF-α or also extended to other cytokines or chemokines. To this end, we quantified levels of CCL3 and CCL5, chemokines known to play important roles in a variety of inflammatory processes [30, 31], in RAW 264.7 cell supernatants. As with TNF-α, fractions 2, 3, and 13 strongly induced production of CCL3 (Fig 7A). Lesser but significant amounts were induced by the original chloroform layer, similar to the effects noted of this layer on production of TNF-α. For CCL5, as shown in Fig 7B, significant increases were again induced by fractions 2, 3, and 13, although it is notable that levels of this chemokine were relatively low. The chloroform layer did not induce a significant increase in the level of CCL5.

CCL3 and CCL5 were also quantified in supernatants from RAW 264.7 cells stimulated with LPS. As shown in Fig 7C, suppression of CCL3 was generally found for the same samples responsible for suppression of TNF-α (samples 6–10 and CL), although in these assays the suppression by fractions 8 and 10 did not reach a statistical level of significance. Similarly, the same set of samples generally inhibited production of CCL5, with fractions 7 and 8 and the chloroform layer displaying significant inhibition. Interestingly, we also noted that fractions 2 and 3 strongly inhibited production of CCL5, suggesting that additional suppressive compounds exist in these fractions whose activity only becomes “visible” when the level of stimulation is low. Overall, our experiments suggest that the stimulatory and inhibitory effects of the chloroform layer and fractions are not specific for production of TNF-α and likely represent more general effects on the secretory products of RAW 264.7 cells. On the other hand, the CCL5 assays further highlight the complexity of the extract and even the fractions collected from the silica gel column. Dominant stimulatory or inhibitory compounds may be masking opposing activities and the activity of these opposing compounds will not be revealed until the dominant compounds are removed or the activities can be separated.

### The role of bacterial endophytes in stimulatory activity of *E*. *purpurea*

Tamta *et al*. [11] and Pugh et. al. [13] have previously shown that macrophage stimulatory activity of *E*. *purpurea* extracts can arise from LPS and lipoproteins of bacterial origin. Based on these reports, we employed the Chromo-LAL assay to measure the quantity of bacterial LPS in the original ethanolic *E*. *purpurea* extract. The extract was found to contain LPS at a concentration of 14.6 ± 8.4 EU/mg (where 14.6 represents the mean of two independent experiments, each performed in triplicate (N = 6) and 8.4 is the standard deviation associated with these measurements). As indicated by the poor precision of these measurements, quantification of LPS levels in the ethanol extract proved difficult. Excessive well to well variation was observed in the Chrom-LAL assay of the extract (but not LPS standards), and levels of LPS in the extract were observed to decrease over time with repeated assays. The high well to well variability could be explained by the inhomogeneous nature of the dried ethanol extract, which had a tar-like consistency, making it very difficult to solubilize (even with extensive sonication). There is literature precedent that endotoxin can exist in aggregates, and that the nature of these aggregates dictates biological activity [32]. It is likely that LPS aggregates in the poorly solubilized extract are unevenly distributed among assay wells, giving rise to assay variability.

Although precise measurements of LPS levels in the ethanolic *E*. *purpurea* extract were difficult, our experiments did indicate the presence of bacterial LPS in this extract. Taking these experiments a step further, it was of interest to test the hypothesis that this LPS could result from bacterial endophytes (bacteria that live in the plant without causing overt disease). This hypothesis has previously been proposed by Pasco [12, 13], and was shown to hold for studies with alfalfa plants, but not tested for *Echinacea* plants. To evaluate the role of bacterial endophytes in immunostimulatory activity of *E*. *purpurea*, *E*. *purpurea* plants were grown in a sterile environment from sterilized seeds. At approximately 6 weeks of age, the plants were harvested and extracted with 75% ethanol under aseptic conditions. The plants were small, so we used the entire plant and not just the roots to produce the ethanol extract. The extracts were then tested for LPS levels, and for their ability to stimulate production of TNF-αfrom RAW 264.7 cells. As a negative control to account for LPS introduced though laboratory manipulation, an extract was prepared from an empty vessel to which no plant material was added. As a positive control, this experiment included a plant grown under identical conditions to the sterile plants, but from a seed that had been subjected to surface sterilization only (a process that removes surface bacteria, but preserves endophytes). This positive control plant was selected from among 44 individual *E*. *purpurea* plants cultivated from surface sterilized seeds, and was intentionally chosen because extraction of this plant produced moderately high levels of LPS (plant 49, S1 Table).

**Fig 8:**
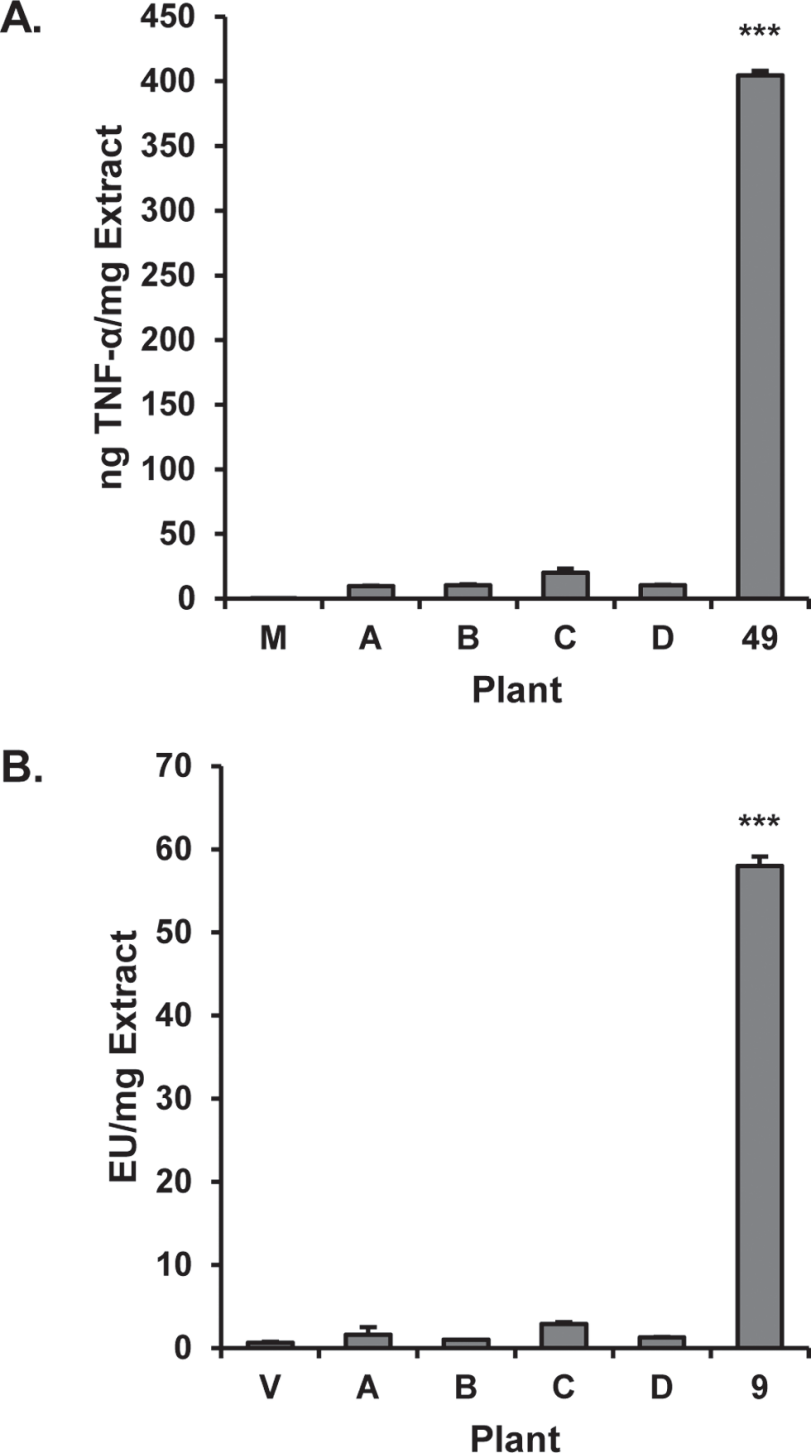
*Echinacea purpurea* plants grown from sterilized seeds. *E*. *purpurea* seeds were sterilized following removal of the epidermis and grown under sterile conditions for six weeks. The negative control was prepared using the same reaction vessels and solvents as for the individual plants, but without plant material. The seed used to grow plant 49 was subjected to surface sterilization but with epidermis intact (see S1 Table). Plants were harvested and extracts tested for LPS content (A) and ability to induce production of TNF-α from RAW 264.7 cells after 16–18 h incubation (B). Data show the means ± SD of duplicate experiments measuring TNF-α secretion in panel A, and the mean ± SEM of triplicate measurements of LPS content in panel B. Statistical analysis was performed using the Student’s T test, ***p < 0.001.

The extracts prepared from sterile seeds, and the negative control, contained very low LPS levels (on the order of the limit of detection, Fig 8). The low levels of LPS observed for the sterilized seeds is supportive of the success of the method employed for sterilization; recent reports from Pasco *et*. *al*. have shown a very strong correlation between levels of bacterial LPS and plant bacterial load [13]. Conversely, LPS was readily detected in the positive control plant. As expected, none of the samples stimulated production of TNF-α from RAW 264.7 cells except the extract prepared from the positive control plant. These data suggest that LPS, originating from endophytic bacteria, can indeed contribute to the stimulatory activity of *E*. *purpurea* extracts. Undoubtedly, as suggested previously [11], additional bacterial components such as Braun type lipoproteins are also involved. Interestingly, earlier studies attributed immunostimulatory activity of *E*. *purpurea* extracts to the presence of polysaccharides [33–35] produced by the *E*. *purpurea* plant. Plant polysaccharides do not appear to be responsible for the stimulatory activity observed in these experiments, given that activity was lost upon seed sterilization. Notably, however, previous studies have shown that polysaccharides are present in some aqueous *Echinacea* extracts, but not in ethanolic extracts such as those evaluated here [36]. The possible role of plant polysaccharides as compared to bacterial components in the activity of aqueous *E*. *purpurea* extracts remains to be evaluated.

## Conclusions

These studies demonstrate the complexity of the modulatory effects of *E*. *purpurea* extracts and their constituents on cytokine production by macrophage-like cells. It is clear that ethanolic extracts contain multiple constituents [including alkylamides, xanthienopyran, and others) that can suppress the production of pro-inflammatory cytokines. However, these activities can be partially or completely masked by stimulatory compounds, including [but likely not limited to) bacterial LPS. Our studies support the hypothesis that the macrophage-stimulatory activity present in *E*. *purpurea* extracts can originate from bacterial endophytes. It is worth noting, however, that in the commercial preparation of *Echinacea* extracts, bacteria (and their associated macrophage-stimulatory compounds) could also come from other sources, such as surface contamination of botanical samples. Finally, our results also demonstrate that, contrary to popular belief in the dietary supplements industry, the use of ethanol as a solvent does not prevent the extraction of LPS and other potentially immunostimulatory compounds from bacteria.

Our findings are highly relevant to the design of effective experiments with botanical medicines in the laboratory and clinic. *In vitro*, even with a single extract and single cell type, our experiments clearly demonstrate that the activity of the complex botanical preparation cannot be predicted on the basis of a few marker compounds. Many more experiments will be required to fully understand the activity of this extract and its constituents towards macrophages. *In vivo*, the situation is undoubtedly more complex, with additional factors such as bioavailability, multiple responding cell types, and differences in host genetics influencing the effectiveness of a complex botanical preparation. It is not surprising that the clinical trials performed with *Echinacea*, using different preparations, different protocols, and with different often superficial endpoint analyses, have yielded conflicting information.

## Supporting Information

S1 Fig ^1^H (above) and ^13^C NMR (below) spectra of xanthienopyran (1) [400 MHz for ^1^H and 100 MHz for ^13^C; MeOD]. (TIF)

S1 Table LPS levels in a series of 45 *E. purpurea* plants grown in a sterile environment for three months from surface-sterilized seeds. A recent publication from the Pasco group indicates that LPS content in *E*. *purpurea* extracts is strongly correlated with bacterial load of the plant (as measured by PCR) [13]. Thus, the presence of LPS in a portion of the extracts can likely be attributed to the presence of bacterial endophytes. Plant # 49 was selected as the positive control for the experiments reported in Fig 8 because of its moderately high LPS content.
(TIFF)
