# Supplementary figures and images for: Ethanolic Echinacea purpurea Extracts Contain a Mixture of Cytokine-Suppressive and Cytokine-Inducing Compounds, Including Some That Originate from Endophytic Bacteria
Source: PLoS One. 2015 May 1;10(5):e0124276. doi: 10.1371/journal.pone.0124276 (PMC4416932; doi:10.1371/journal.pone.0124276)

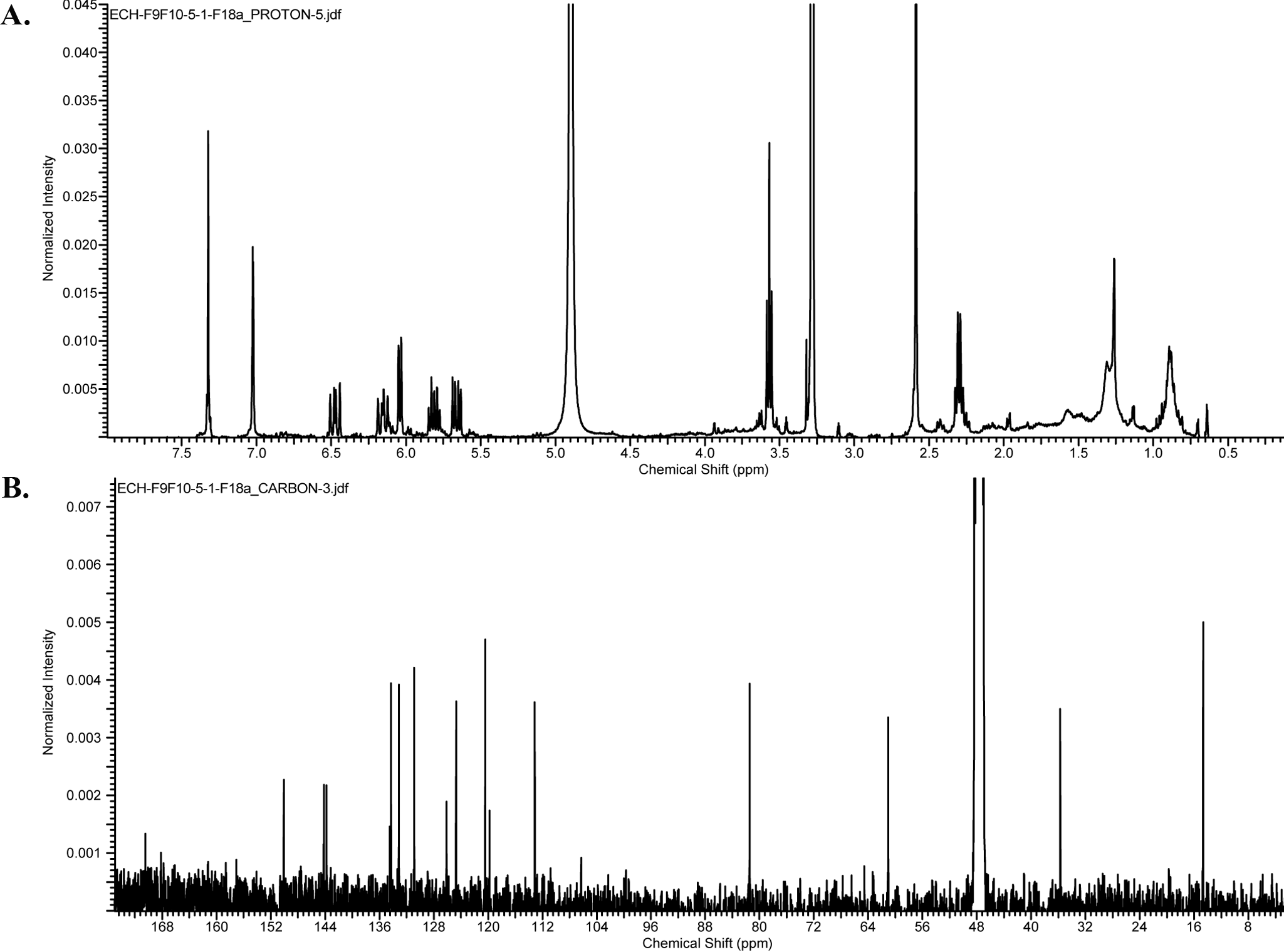

Supplement: S1 Fig — (TIF) [file pone.0124276.s001.tif]
